# A dual-responsive nanocapsule *via* disulfide-induced self-assembly for therapeutic agent delivery†
†Electronic supplementary information (ESI) available: Experimental section, MALDI-TOF-MS spectra of the SNBDP, the changes of the diameter and PDI in PBS with FBS FT-IR of SNBDP NCs before and after light irradiation, illustration of the formation of supramolecular vesicles, cell viability of HeLa cells incubated with SNBDP NCs, representative CLSM images of HeLa cells for 0.5 h without pretreatment and pretreated with 10 mM GSH, the size of RhB@SNBDP NCs measured by DLS, TEM images and fluorescence spectra of RhB@SNBDP NCs and RhB in water, TEM images of the ICG@SNBDP NCs, representative CLSM images of HeLa cells incubated with ICG@SNBDP NCs for 0.5 and 2 h and cell viability after water-bath hyperthermia. See DOI: 10.1039/c5sc03707g


**DOI:** 10.1039/c5sc03707g

**Published:** 2015-11-25

**Authors:** Wenhai Lin, Tingting Sun, Zhigang Xie, Jingkai Gu, Xiabin Jing

**Affiliations:** a State Key Laboratory of Polymer Chemistry and Physics , Changchun Institute of Applied Chemistry , Chinese Academy of Sciences , 5625 Renmin Street , Changchun , Jilin 130022 , P. R. China . Email: xiez@ciac.ac.cn ; Fax: +86 431 85262775 ; Tel: +86 431 85262775; b University of Chinese Academy of Sciences , Beijing 100049 , P. R. China; c Research Center for Drug Metabolism , College of Life Sciences , Jilin University , Changchun 130012 , P. R. China

## Abstract

One-step synthesis of fluorescent molecules (SNBDP) containing one disulfide bond and two *o*-nitrobenzyl groups was demonstrated *via* multi-component Passerini reaction. This hydrophobic SNBDP could self-assemble into nanocapsules (SNBDP NCs) in aqueous solution *via* disulfide-induced assembly. The obtained nanocapsules were stable in aqueous solution for several weeks and exhibited enhanced fluorescence when nanocapsules were destroyed due to disaggregation-induced emission. The nanocapsules not only were reduction-sensitive and light-responsive, but also could be endocytosed by HeLa cells for cellular imaging. The enhanced fluorescence in the glutathione (GSH) pretreated HeLa cells showed that the compound was reduction-sensitive in living cells. *In vitro* WST-8 assays showed the nanocapsules were biocompatible and could further be used as drug delivery carriers. Indocyanine green (ICG), a clinically approved NIR dye, was loaded into the nanocapsules (ICG@SNBDP NCs). ICG@SNBDP NCs showed enhanced photothermal efficacy compared with same concentration of free ICG under 808-nm laser irradiation. Consequently, ICG@SNBDP NCs upon NIR irradiation can effectively kill cancer cells through local hyperthermia. These results highlight the potential of disulfide-induced nanocapsules as smart nanoparticles for cellular imaging and therapeutic agent delivery.

## Abstract 2

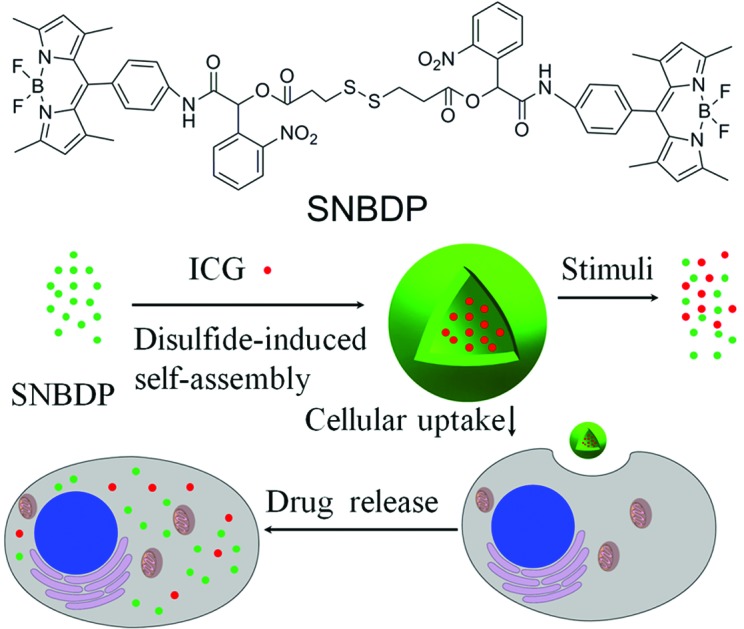
One-step synthesis of fluorescent molecules (SNBDP) containing one disulfide bond and two *o*-nitrobenzyl groups was demonstrated *via* multi-component Passerini reaction.

## Introduction

Self-assembly of small molecules (SASM) is a fascinating and useful method to fabricate various functional nanomaterials. Compared with macromolecular counterparts, SASM has shown great advantages because of precise molecular structure and repeatable large-scale synthesis.^1^,^2^ Programmed SASM could be achieved by using different supramolecular chemistry, such as hydrophobic interactions, electrostatic interactions, π–π interactions and hydrogen-bond interactions.^3^,^4^ However, development of SASM is relatively slow compared to the self-assembly of polymers.^5^–^7^ Up to now, SASM is mainly realized by choosing amphiphilic small molecules or π-conjugated monomers.^8^–^10^ For example, Yan *et al.* have reported a nanodrug from assembly of an amphiphilic drug–drug conjugate for cancer therapy.^11^ Our previous work has demonstrated that unadulterated BODIPY (4,4-difluoro-4-bora-3*a*,4*a*-diaza-*s*-indacene) dimer could self-assemble into fluorescent nanoparticles for cellular imaging because of its π-conjugated structure.^12^ Unfortunately, most drugs and dyes are hydrophobic and are not easily modified by hydrophilic molecules, and cannot self-assemble into nanoparticles. It is thus desired to develop some new ways to make functional nanomaterials. Very recently, nanomedicines prepared *via* disulfide bond bridges have been demonstrated and caught our attention.^13^ Disulfide-induced nanomedicines (DSINMs) have been promoted and stabilized by the insertion of a single disulfide bond into hydrophobic molecules, in order to balance the competition between intermolecular forces. However, it is not clear whether this disulfide-induced assembly is universal for other hydrophobic molecules. It is well-known that hydrophobic small molecules without π-conjugated structures can not self-assemble into stable nanoparticles. Up to now, no work on disulfide-induced fluorescent nanoparticles has been reported.

Compared to traditional fluorescent organic dyes, fluorescent nanoparticles show unique chemical and optical properties, such as brighter fluorescence, higher photostability and better biocompatibility, for their application in bioimaging and diagnostics.^14^–^17^ Moreover, fluorescent nanoparticles could provide a versatile platform for loading drugs or bioactive molecules. These properties make them useful in medical and biological fields.^18^–^20^ Ideally fluorescent nanoparticles should possess super brightness, excellent photostability and good biocompatibility.^21^,^22^

BODIPY (BDP) dyes have drawn much attention due to their excellent photophysical properties and been widely used in cellular imaging and chemical sensors.^23^,^24^ It has been reported that fluorescent BDP could be adapted to multi-component reaction chemistry with high fluorescence quantum yields and good cell permeability for *in vivo* imaging of phagocytic macrophages.^25^ However, BDP derivatives cannot directly self-assemble into nanostructures in aqueous solution. It is still a great challenge to prepare fluorescent nanoparticles from versatile BDP dyes.

**Scheme 1:**
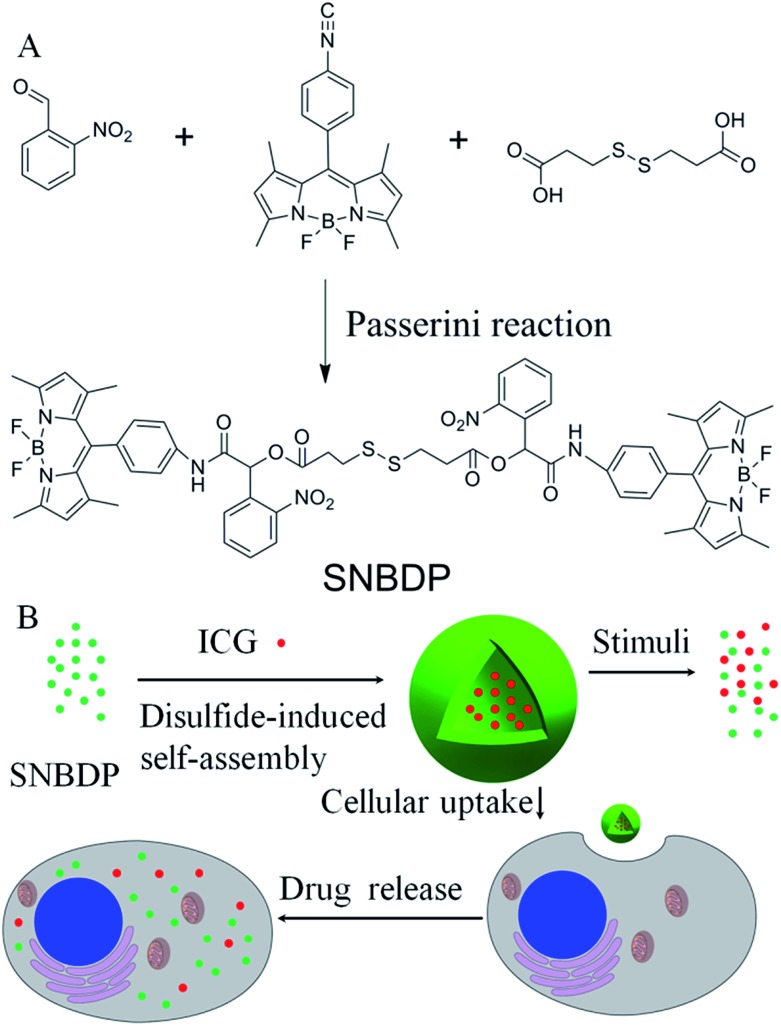
(A) Synthesis of the SNBDP *via* multi-component Passerini reaction. (B) A schematic illustration of the self-assembly, disassembly and cellular uptake of SNBDP NCs.

In this report, a dual-responsive fluorescent molecule (SNBDP, Scheme 1A) was synthesized by way of a one-pot multi-component Passerini reaction from 4,4-difluoro-8-(4-isocyanophenyl)-3,5-dimethyl-4-bora-3*a*,4*a*-diaza-indacene (NC-BDP), 3,3-dithiodipropionic acid and *o*-nitrobenzaldehyde. SNBDP could self-assemble into nanocapsules (SNBDP NCs) by virtue of the disulfide bond bridge. The self-assembly and stimuli-induced disassembly behaviours of SNBDP NCs were then investigated in detail. Furthermore, SNBDP NCs could be internalized and disassembled to emit green fluorescence for bioimaging. ICG were loaded into the NCs to investigate their potential applications for drug delivery (Scheme 1B).

## Results and discussion

### Synthesis and characterization of NCs

Three-component Passerini reaction could combine isocyanides, aldehydes and carboxylic acids *via* ester and amide linkages in an atom-economic way. This reaction has been employed to synthesize diverse monomers and functional polymers under mild conditions in the absence of catalysts with high tolerance to many functional groups.^26^–^29^ We have reported on reduction-sensitive amphiphilic copolymers for drug delivery^30^ and cross-linked polymers for photocatalysis *via* Passerini reaction.^31^ In this work, multi-component Passerini reaction was used to synthesize the target dye molecules.

**Fig. 1:**
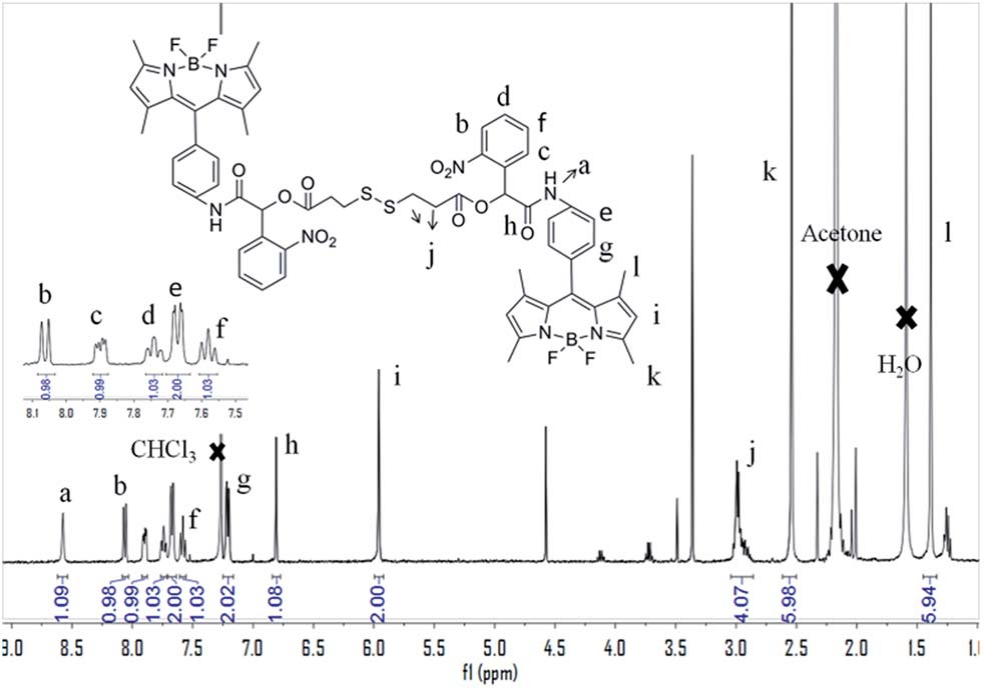
^1^H NMR characterization of the SNBDP.

First, 4,4-difluoro-8-(4-isocyanophenyl)-3,5-dimethyl-4-bora-3*a*,4*a*-diaza-indacene (NC-BDP) was synthesized according to the literature methods.^25^ Then NC-BDP, 3,3-dithiodipropionic acid and *o*-nitrobenzaldehyde were mixed in dichloromethane and stirred at room temperature for 4 days (Scheme 1A). After purification on a silica gel column, SNBDP was obtained and characterized by proton nuclear magnetic resonance (^1^H NMR) and matrix-assisted laser desorption/ionization time-of-flight mass spectrometry (MALDI-TOF MS). As shown in Fig. 1, all the protons corresponding to the compound could be clearly resolved in the ^1^H NMR spectrum, and the integration ratios of these peaks agreed well with that of theoretical calculation. More importantly, the signals at 8.57 and 6.76 ppm for imine and methine protons indicated that NC-BDP was successfully incorporated into SNBDP through Passerini reaction. The peak at *m*/*z* 1210.4 in the MALDI-TOF MS spectrum is close to the theoretical molecular weight of SNBDP (Fig. S1†), further confirming the successful synthesis of SNBDP.

**Fig. 2:**
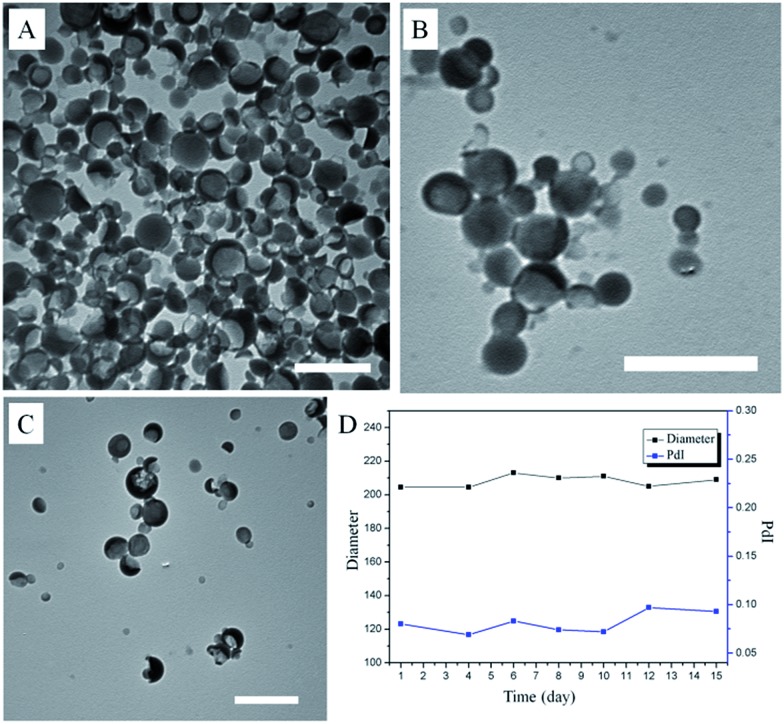
(A) and (B) TEM images of SNBDP NCs. (C) TEM images of SNBDP NCs after storing for a month. (D) The changes of the diameter and PDI with different times measured by DLS. Scale bar, 500 nm.

Interestingly, SNBDP could self-assemble into nanocapsules (SNBDP NCs) in aqueous solution *via* a simple nano-precipitation method. Briefly, a tetrahydrofuran (THF) solution of SNBDP was added into water under stirring and then dialyzed to remove THF. The morphology and size distribution of SNBDP NCs were characterized by transmission electron microscopy (TEM) and dynamic light scattering (DLS), respectively. The TEM image in Fig. 2A showed a typical vesicular structure with an average diameter of about 200 nm. The partially collapsed morphologies further confirmed their vesicular structure in Fig. 2B. The morphologies of SNBDP NCs were retained even after storing for one month (Fig. 2C). Moreover, the diameter and the polydispersity index (PDI) measured by DLS almost remained unchanged over two weeks (Fig. 2D). These results demonstrated that SNBDP NCs were stable in aqueous solution. As shown in Fig. 2D, the average diameter of SNBDP NCs measured by DLS was 204 nm, which was consistent with that observed by TEM. SNBDP NCs also exhibited favorable structural stability in physiological environment as evidenced by the unaltered particle size and PDI observed by incubating the NCs in PBS (pH 7.4) containing FBS (10%) at 37 °C for up to 24 h (Fig. S2†).

**Fig. 3:**
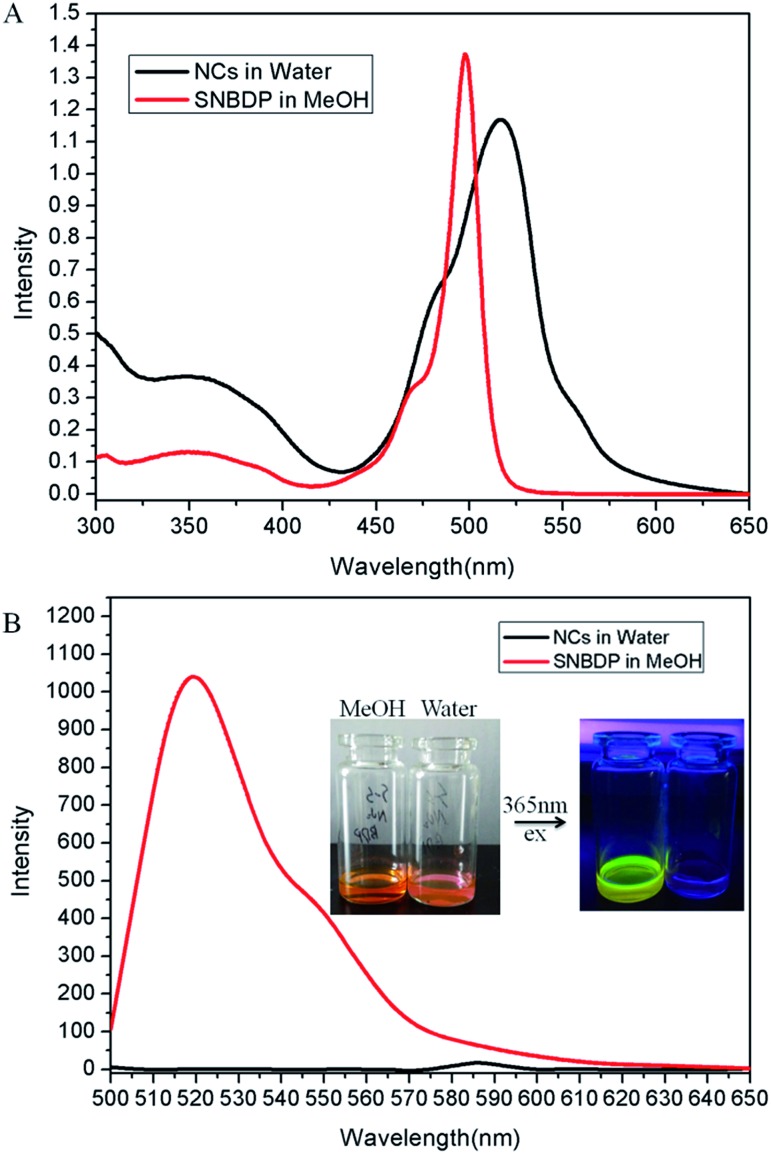
(A) UV-vis absorption and (B) fluorescence spectra of SNBDP NCs in water and the SNBDP in MeOH.

The optical properties of the SNBDP NCs and SNBDP were studied by UV-vis absorption and photoluminescence spectra. As shown in Fig. 3, although both of them exhibited similar absorption spectra, the maximum absorption band of SNBDP NCs in water was centred at 516 nm, which was red-shifted by 20 nm relative to that of SNBDP in MeOH (Fig. 3A). The bathochromic shift absorption band of NCs indicated a J-aggregate of SNBDP in aqueous solution, which is consistent to reported data for aggregation of BODIPY.^32^,^33^ The maximum fluorescence wavelength was seen at 520 nm for SNBDP, but almost no fluorescence was observed for SNBDP NCs due to aggregation-caused quenching (ACQ) (Fig. 3B). To visually observe the phenomenon of ACQ,^34^ photos of SNBDP NCs and SNBDP were taken under 365-nm light irradiation. As shown in Fig. 3B, SNBDP in MeOH emitted a strong yellow–green fluorescence while no fluorescence was observed for SNBDP NCs.

For the vesicular aggregates, a possible mechanism is presented based on the results of calculated electron density distribution. The optimized ground-state geometrical structure was optimized using density functional theory (DFT) at B3LYP/6-31G* level.^35^–^37^ The calculated electron density distribution was interesting. BDP fragments on the ends of the molecule carried a positive charge (+0.60 e) and the central S–S fragments carried negative charge (–0.60 e). Therefore, we predict that the major reason of the formation of the nanoparticles was electrostatic forces as shown in Fig. S3A.† As shown in Fig. S3B,† the J-type arrangement of SNBDP units first formed single layers through the electrostatic interactions, and then vesicles were obtained *via* hydrophobic and π–π interactions between different layers of SNBDP. Some similar vesicular aggregates have been reported using BODIPY molecules.^38^,^39^ Transition behavior from small molecules to supramolecular capsules has been covered in a recent review.^40^ It is thus not surprising that nanocapsules are observed in this work.

### Dual-responsive behaviours of NCs

**Fig. 4:**
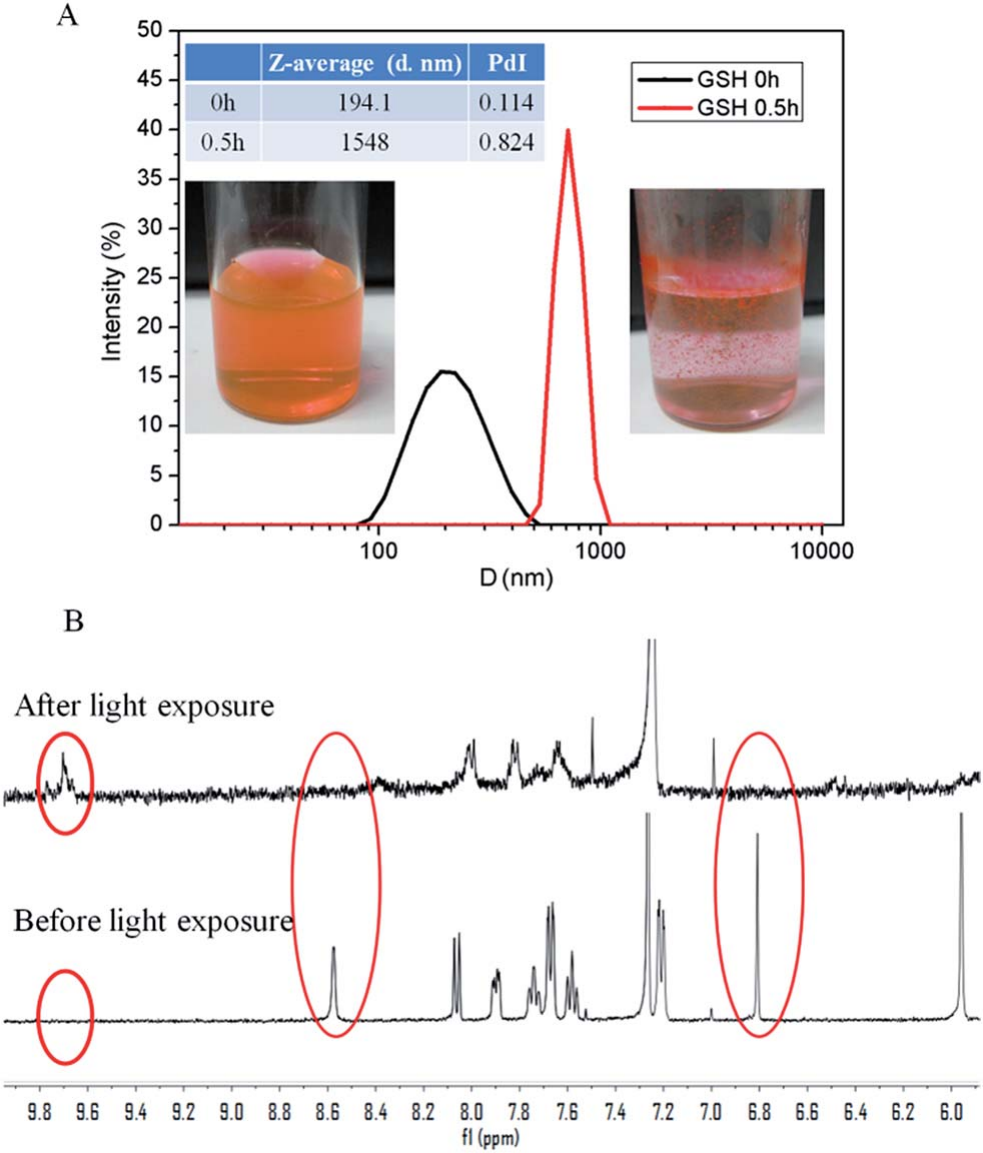
(A) Reduction-induced disassembly resulting in size changes of SNBDP NCs. (B) The magnetic resonance spectra after and before light exposure.

The reduction-induced dissociation of disulfide bonds in SNBDP NCs was monitored by DLS in the presence of 10 mM GSH (Fig. 4A). An increase in the diameter of SNBDP NCs from 194.1 to 1548 nm was observed after 30 min. The PDI also increased from 0.114 to 0.824, which confirmed the disassembly of SNBDP NCs. The solution changed from clear to cloudy and a large number of precipitates were found (Fig. 4A, right photo). The formation of precipitates is attributed to the disassembly of SNBDP NCs, resulting in the rearrangement of hydrophobic fragments into large agglomerates.^41^ These results demonstrate SNBDP NCs are reduction-sensitive and could be destroyed in the presence of reducing agents.

Owing to their non-invasiveness and the possibility of remote spatiotemporal control, a large variety of photoresponsive systems has been engineered in the past few years.^42^,^43^ *o*-Nitrobenzyl and its derivatives have been widely used as photocleavable groups in drug release.^44^ Upon exposure to 365 nm light, effective photolysis of SNBDP NCs was evidenced by ^1^H NMR (Fig. 4B) and Fourier-transform infrared (FT-IR) spectroscopy (Fig. S4†). The characteristic signals at 8.57 and 6.76 ppm disappeared upon exposure to light irradiation (Fig. 4B). Moreover, the appearance of a signal at 9.73 ppm indicated the SNBDP was cleaved into carbonyl photoproducts. The photolysis was further corroborated by FT-IR spectroscopy where peaks at 3400 cm^–1^ (broad) and 1740 cm^–1^ appeared in the spectrum of the irradiated sample, indicating of the formation of photoproducts containing hydroxyl and carbonyl groups (Fig. S4†).

### Intracellular uptake and bioimaging of SNBDP NCs

The biocompatibility of nanomaterials is very important for their biomedical applications. The *in vitro* cytotoxicities of SNBDP NCs toward human cervical carcinoma (HeLa) cells were evaluated by cell counting kit-8 (CCK-8).^45^,^46^ Fig. S5† showed the viability of cells incubated with various concentrations of SNBDP NCs for 24 h. SNBDP NCs did not exhibit obvious inhibition effects on cell proliferation for concentrations up to 50 μg mL^–1^ (Fig. S5†).

**Fig. 5:**
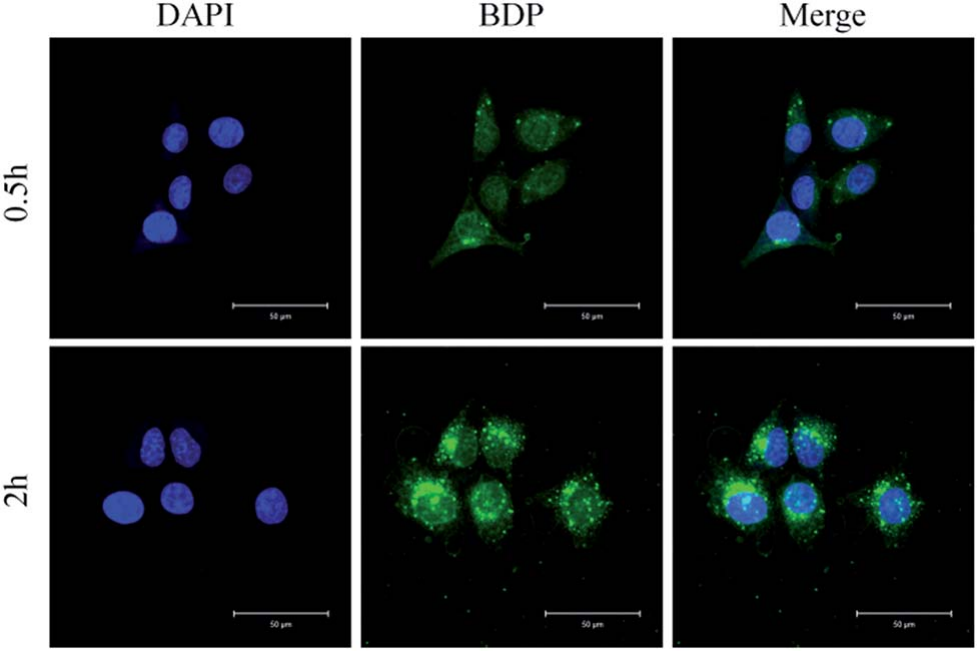
Representative CLSM images of HeLa cells incubated with SNBDP NCs for 0.5 and 2 h. For each panel, the images from left to right show cell nuclei stained by DAPI (blue), BDP fluorescence in cells (green), and overlays of both images. Scale bar, 50 μm.

The cellular uptake was evaluated in HeLa cells by confocal laser scanning microscopy (CLSM). HeLa cells were incubated with 10 μg mL^–1^ of SNBDP NCs for 0.5 and 2 h. The cell nuclei were stained with 4′,6-diamidino-2-phenylindole (DAPI). The intensity of green fluorescence increased upon prolonging the incubation time from 0.5 to 2 h (Fig. 5), indicating that SNBDP NCs could be internalized and located in the cytoplasm. It is noteworthy that some green fluorescence could be seen in nuclei after 2 h incubation, showing that SNBDP NCs or BDP could escape from endosomes. It is well known that endosomal escape is important and necessary for nanomedicine.^47^ The reduction-responsive SNBDP NCs were assessed in the intracellular environment. HeLa cells were pretreated with 10 mM GSH according to the reported protocol,^48^ and then incubated with SNBDP NCs for 0.5 h. The cells without pretreatment of GSH were used as controls. As expected, stronger green fluorescence was observed in cells pretreated by GSH (Fig. S6†). As reported previously, GSH pretreatment did not significantly affect the endocytosis ability of cells.^49^ Thus it could be inferred that the enhanced fluorescence intensity was ascribed to the rapid disassembly of SNBDP NCs.

**Fig. 6:**
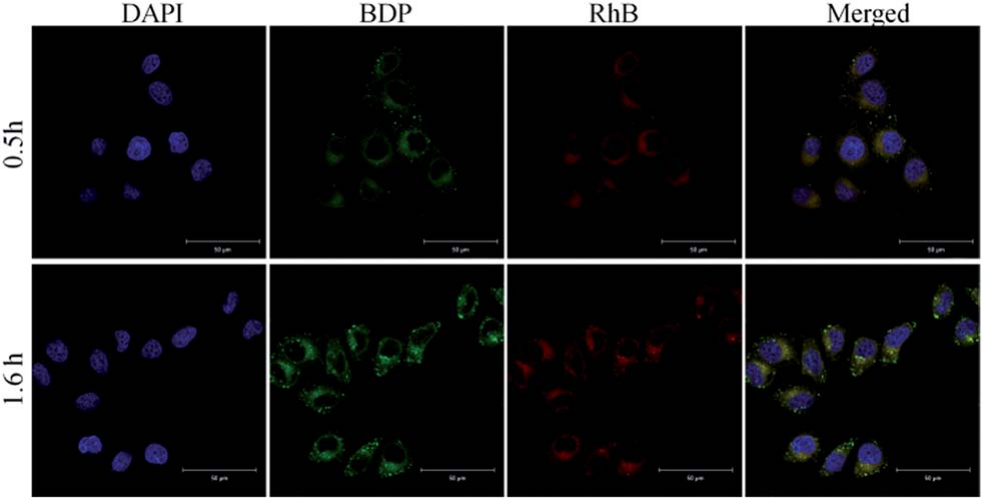
Representative CLSM images of HeLa cells incubated with RhB@SNBDP NCs for 0.5 and 1.6 h. For each panel, the images from left to right show cell nuclei stained by DAPI (blue), BDP fluorescence (green), RhB fluorescence (red) and overlays of the images. Scale bar, 50 μm.

In order to demonstrate the potential of SNBDP NCs as drug carriers, rhodamine B (RhB) as a model drug was loaded into SNBDP NCs. The RhB-loaded NCs (RhB@SNBDP NCs) were prepared by adding a THF solution of SNBDP into RhB solution. The average diameter of RhB@SNBDP NCs was 230.3 nm, which was slightly larger than that of SNBDP NCs because of introduced RhB (Fig. S7†). The fluorescence spectra of RhB@SNBDP NCs exhibited a red shift compared to RhB because of the aggregation of RhB in NCs (Fig. S8†). The cellular uptake was evaluated in HeLa cells by CLSM (Fig. 6). The red fluorescence of RhB was colocalized exactly with the green fluorescence of BDP, which confirmed that SNBDP NCs could encapsulate hydrophilic cargos and transport them (such as organic dyes and anti-cancer drugs) into the cells.

### ICG-loaded SNBDP NCs

As mentioned above, SNBDP NCs could be potentially used as drug carriers to deliver therapeutic agents. Herein, ICG was chosen as a typical drug to study the drug delivery behaviour. ICG-loaded NCs (ICG@SNBDP NCs) were prepared as below. A THF solution of SNBDP was added into an aqueous solution of ICG, and the mixture was stirred, then dialyzed to remove THF and free small molecules. When an ICG content of 20 wt% was used in the feed, ICG-loaded NCs (ICG@SNBDP NCs) with loading efficiency of 46.3% were obtained. The content and concentration of ICG was calculated to be 9.2 wt% and 11.1 μg mL^–1^ according to the standard curve, respectively.

**Fig. 7:**
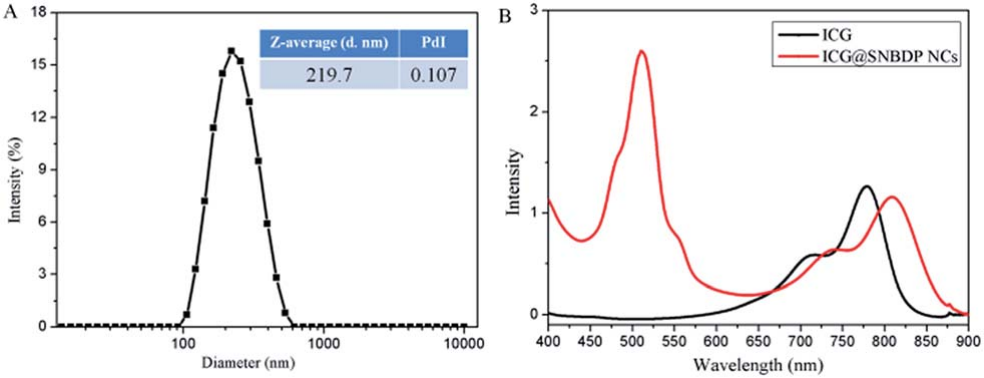
(A) The size and PDI of the ICG@SNBDP NCs. (B) UV-vis absorption of ICG and the ICG@SNBDP NCs.

The size and morphology of ICG@SNBDP NCs were studied by DLS (Fig. 7A) and TEM (Fig. S9†), respectively. The average diameter of ICG@SNBDP NCs was 219.7 nm and the PDI was 0.107. The size of ICG@SNBDP NCs was bigger than that of SNBDP NCs because of the loading of ICG. Enhanced contrast for ICG@SNBDP NCs as revealed by TEM (Fig. S9†) confirmed the successful encapsulation of ICG. The optical properties of the ICG@SNBDP NCs were recorded by UV-vis spectrometry. The maximum absorption of ICG@SNBDP NCs exhibited a red shift compared with free ICG because of the aggregation of ICG, which also confirmed that ICG was encapsulated into SNBDP NCs (Fig. 7B).

**Fig. 8:**
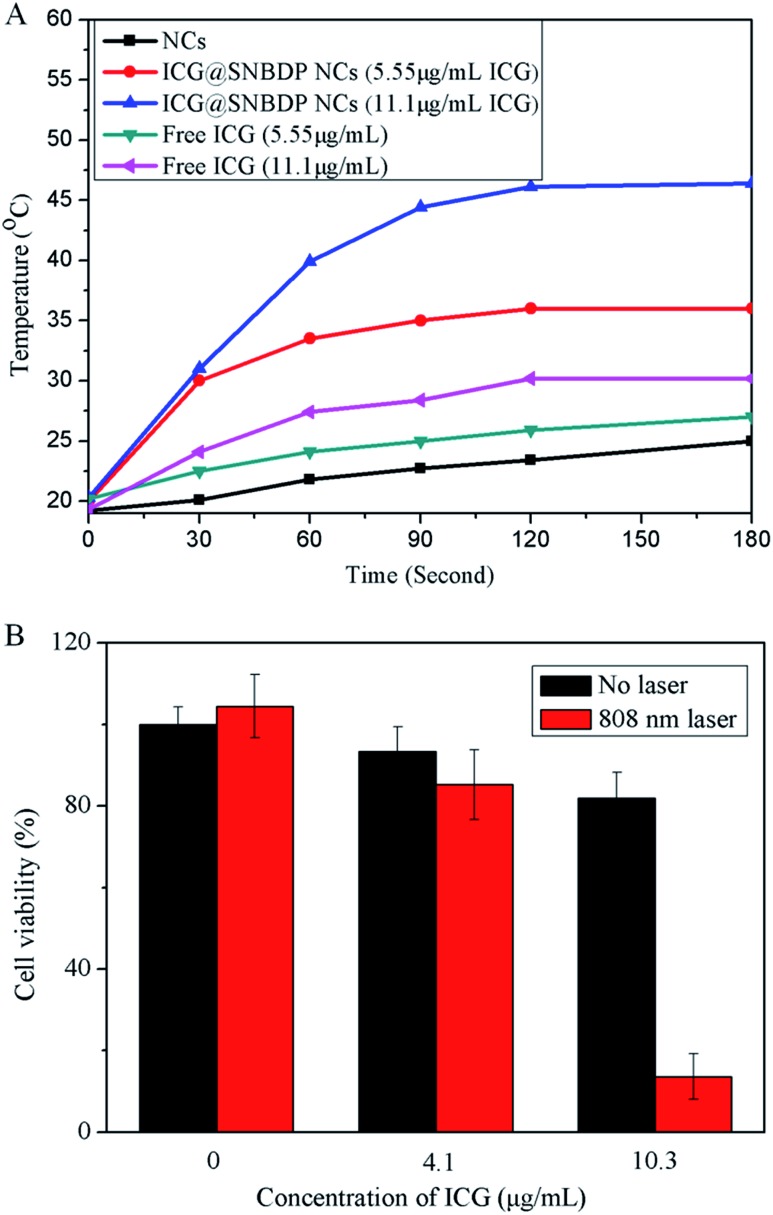
(A) Heating curves of the ICG@SNBDP NCs and free ICG under NIR laser irradiation with different concentrations. (B) Relative cell viabilities of HeLa cells incubated with different concentrations of ICG@SNBDP NCs without or with laser irradiation (808 nm, 2.0 W cm^–2^, 5 min). All the results were repeated three times and presented as mean ± SD.

ICG has been demonstrated to be an effective photothermal agent.^50^ The temperature of ICG@SNBDP NCs solution with ICG content of 11.1 μg mL^–1^ increased from 20.3 to 46.4 °C upon irradiation using a 808-nm laser with intensity of 1 W cm^–2^ for 3 min (Fig. 8A). The temperature reached 36 °C when the concentration of ICG in NCs was 5.55 μg mL^–1^. As a control, the SNBDP NCs solution showed little increase of temperature at the same condition. It was of note that the temperature of free ICG solution only reached 30.2 °C at a concentration of 11.1 μg mL^–1^, which was much lower than that of ICG@SNBDP NCs. The enhanced photothermal effect was ascribed to the aggregation of ICG in NCs. These results indicated the great potential of nanoparticle-based photothermal agents. Many nanoparticles encapsulating ICG indicated the enhanced photothermal effect because of aggregation of ICG. The main reason for using stimuli-responsive NCs is based on this requirement for cancer treatment *in vivo*. Moreover, controlled release and biodegradation are very important for drug delivery system. NCs burst and degrade controllably upon stimuli-responsive behavior as is required after photothermal therapy.

Then the cellular uptake of ICG@SNBDP NCs was evaluated in HeLa cells by CLSM (Fig. S10†). The green fluorescence of BDP was clearly seen in the cytoplasm, which confirmed that the ICG-loaded NCs could be internalized into cells. Then we tested water-bath hyperthermia;^51^ HeLa cells were exposed to 5 min of water-bath hyperthermia (45 and 50 °C), which did not lead to a significant increase in cancer cell death (Fig. S11†). Finally, photothermal therapy experiments towards HeLa cells were carried out. HeLa cells were incubated with different concentrations of ICG@SNBDP NCs for 5 h and then irradiated by the 808-nm laser for 5 min. The cells were incubated for another 18 h, then the cell viabilities were measured by CCK-8 method. More than 80% of cells were killed after laser irradiation with the concentration of ICG at 10.3 μg mL^–1^ (Fig. 8B). In contrast, cells without ICG@SNBDP NCs incubation or laser irradiation did not show obvious death. These results demonstrated that ICG@SNBDP NCs were effective in photothermal activity for cancer therapy.

## Conclusions

This work emphasizes that small molecule based nanocapsules are promoted by the insertion of a single disulfide bond into hydrophobic molecules, and the potential of using multi-component Passerini reaction to develop stimuli-sensitive materials. The DSINM technology, a new way of self-assembly, extends our knowledge of self-assembly and will stimulate the development of functional nanoparticles in various fields. Much work should be done to verify that the DSINM is a universal method for small molecules to self-assemble into nanoparticles. Secondly, multi-component reactions with isocyanides such as the Passerini reaction and Ugi reaction show great potential to synthesize multi-functional molecules under mild conditions.

## Supplementary Material

Supplementary information
