# Supplementary material for: A dual-responsive nanocapsule via disulfide-induced self-assembly for therapeutic agent delivery
Source: Chem Sci. 2015 Nov 25;7(3):1846–52. doi: 10.1039/c5sc03707g (PMC5965061; doi:10.1039/c5sc03707g)
Supplement: Supplementary file 1 [file SC-007-C5SC03707G-s001.pdf]

Electronic Supplementary Material (ESI) for Chemical Science.
This journal is © The Royal Society of Chemistry 2015

Electronic Supplementary Information

A dual-responsive nanocapsule *via* disulfide-induced self-assembly for therapeutic agent delivery

Wenhai Lin,[ab] Tingting Sun,[ab] Zhigang Xie,[a*] Jingkai Gu[c] and Xiabin Jing[a]

[a]State Key Laboratory of Polymer Chemistry and Physics, Changchun Institute of Applied Chemistry, Chinese Academy of Sciences, 5625 Renmin Street, Changchun, Jilin 130022, P. R. China. E-mail: xiez@ciac.ac.cn; Fax/Tel: +86 431 85262775

[b]University of Chinese Academy of Sciences, Beijing 100049, P. R. China

[c]Research Center for Drug Metabolism, College of Life Sciences, Jilin University, Changchun 130012, P. R. China

Content

1. Experimental section

2. Fig. S1 MALDI-TOF-MS spectra of the SNBDP.

3. Fig. S2 The changes of the diameter and PdI with different times measured by DLS in PBS with FBS (10 %).

4. Fig. S3 (A) Ground-state geometrical structure and calculated electron density distribution of SNBDP. (B) Illustration of the formation of supramolecular vesicles.

5. Fig. S4 FT-IR of SNBDP NCs before and after light irradiation.

6. Fig. S5 Cell viability of HeLa cells incubated with SNBDP NCs.

7. Fig. S6 Representative CLSM images of HeLa cells incubated with SNBDP NCs for 0.5 h without pretreatment and pretreated with 10 mM GSH.

8. Fig. S7 (A) The size of RhB@SNBDP NCs measured by DLS. (B) TEM images of the RhB@SNBDP NCs. Scale bar, 1 μm

9. Fig. S8 Fluorescence spectra of RhB@SNBDP NCs and RhB in water.

10. Fig. S9 TEM images of the ICG@SNBDP NCs. Sacle bar, 500 nm.

11. Fig. S10 Representative CLSM images of HeLa cells incubated with ICG@SNBDP NCs for 0.5 h and 2 h.

12. Fig. S11 Relative cell viabilities of HeLa cells after water-bath hyperthermia at 45 ^{o}C and 50 ^{o}C in 5 minutes. all the results were repeated three times and presented as mean ± SD.

Experimental section

Materials. Glutathione (GSH) was purchased from Shanghai Yuanye Biological Technology Co., Ltd.. 3,3-dithiodipropionic acid and *o*-nitrobenzaldehyde were purchased from Shanghai Sun Chemical Technology Co., Ltd.. Chloroform-d ($CDCl_3$) was purchased from Qingdao Tenglong Weibo technology Co., Ltd.. 4,4-Difluoro-8-(4-isocyanophenyl)-3,5-dimethyl-4-bora-3a,4a-diaza-s-indacene (NC- BDP) was synthesized according to literature methods.[24] All reagents were purchased from commercial sources and used without further treatment, unless indicated otherwise.

Characterizations. ^1H NMR spectra were measured in $CDCl_3$ at room temperature by an AV-400 NMR spectrometer from Bruker. Size and size distribution of the NCs were determined by Malvern Zeta-sizer Nano for dynamic light scattering (DLS). The measurement was carried out at 25 ^oC and the scattering angle was fixed at 90°. The morphology of the NCs was measured by transmission electron microscopy (TEM) performed on a JEOL JEM-1011 electron microscope operating at an acceleration voltage of 100 kV. To prepare specimens for TEM, a drop of NCs solution (0.1 mg/mL) was deposited onto a copper grid with a carbon coating. The specimens were air-dried and measured at room temperature. The calculations of ground-state geometrical structure and electron density distribution performed by Gaussian 09 program.[S1]

Synthesis of the SNBDP. A mixture of NC-BDP (34.9 mg, 0.1 mmol), 3,3-dithiodipropionic acid (10.5 mg, 0.05 mmol) and *o*-nitrobenzaldehyde (15.1 mg, 0.1

mmol) in CH_2Cl_2 (0.5 mL) was stirred at room temperature for 4 days. Finally, the reaction mixture was chromatographed on a silica gel column (CH_2Cl_2:EtOAc = 10:1) to yield 15 mg SNBDP (yield: 25%).

Preparation of SNBDP NCs. The nano-precipitation method was used to prepare NCs. The SNBDP (3 mg) was dissolved in THF (5 mL). After stirred about 5 min, the solution was added dropwise to deionized water (10 mL) and stirred to evaporate the organic solvent followed by dialysis for 24 h.

Preparation of the RhB@SNBDP NCs and ICG@SNBDP NCs. the experiment was similiar to the preparation of NCs. Just the deionized water were changed to the ICG or RhB aqueous solution.

Reduction- and light-responsive behaviours of SNBDP NCs. For reductive degradation, the SNBDP NCs (200 μg/mL) were added with 10 mM GSH under shake at 37 ^{o}C for 0.5 h, then were measured by DLS. As for photo-degradation, the SNBDP NCs (200 μg/mL) were stirred under light exposure with a Hg-Xe tool adjusted at 365 nm for 2 h. The photoproducts were freeze-dried for measurements.

Cell uptake studies. The cellular uptake and intracellular disassembly behaviors of SNBDP NCs were determined by confocal laser scanning microscopy (CLSM) toward HeLa cells. The cells were seeded in 6-well plates at about 200,000 cells per well in 1.5 mL Dulbecco Modified Eagle Medium (DMEM, GIBCO) containing 10% fetal bovine serum, supplemented with 100 U/mL penicillin and 100 U/mL streptomycin, and incubated at 37 ^{o}C in 5 % CO_2 atmosphere for 24 h. After removing

culture medium, the cells were then treated with 10 mM GSH for 2 h. Then the cells were washed with PBS and incubated at 37 °C for additional 0.5 h with SNBDP NCs. Cells without GSH treatment were used as the control. Then the culture medium was removed and cells were washed with PBS three times. Thereafter, the cells were fixed with 4% formaldehyde for 10 min at room temperature, and the cell nuclei were stained with 4 ,6-diamidino-2-phenylindole (DAPI, blue). CLSM images of cells were obtained with an Olympus FluoView 1000 CLSM. In addition, the cells was incubated with NCs for 0.5 h and 2 h without GSH pretreated to evaluate the uptake and gather in the cytoplasm of NCs. The same experiments were done with the RhB@SNBDP NCs and ICG@SNBDP NCs.

Cell viability assays. The biocompatibility of NCs was assessed with a 4-[3-(4-iodophenyl)-2-(4-nitrophenyl)-2*H*-5-tetrazolio]-1,3-benzene disulfonate (WST) assay kit (CCK-8) against HeLa cells.[44, 45] The cells were seeded in 96-well plates at about 10000 cells per well in 100 μL DMEM, and incubated at 37 °C in 5% CO_2 atmosphere for 24 h. After removing culture medium, SNBDP NCs diluted in DMEM (100 μL) were added to cell wells with various concentrations from 10 to 50 μg/mL. The cells were incubated for another 24 h. Then 10 μL of CCK-8 was added to cell wells and cells were incubated for another 4 h. The absorbance of the solution was measured on a Bio-Rad 680 microplate reader at 450 nm. Cell viability (%) was calculated based on the following equation: (Asample/Acontrol) × 100 %, where Asample and Acontrol denote as absorbencies of the sample well and control well, respectively.

Photothermal Experiments. To study the photothermal property of the ICG@SNBDP NCs with different concentrations were irradiated under an 808 nm laser at 1 W/cm^2 for 3 min with a temperature detector used to monitor their temperature.

Cell Ablation Study. Cells harvested in a logarithmic growth phase were seeded in 96-well plates at a density of 10^5 cells per well and incubated in DMEM for 24 h. The medium was then replaced by ICG@SNBDP NCs at various concentrations and incubated for 5 h. After that, cells were irradiated by the 808 nm laser at a power of 2 W/cm^2 for 5 min. After laser irradiation, the medium was replaced by DMEM (100 μL) and the cells were incubated for another 18 h, followed by WST-8 assays to quantitatively measure the relative cell viabilities.

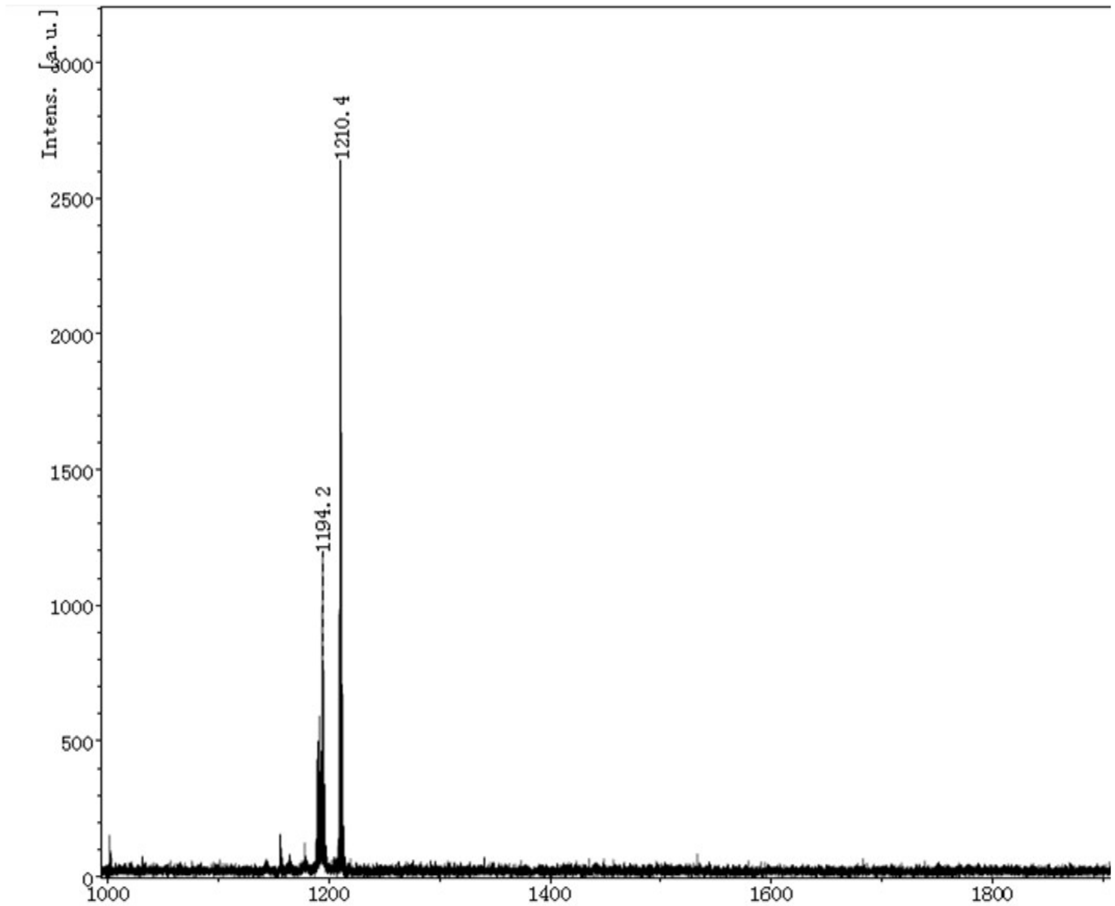

Fig. S1 MALDI-TOF-MS spectra of the SNBDP.

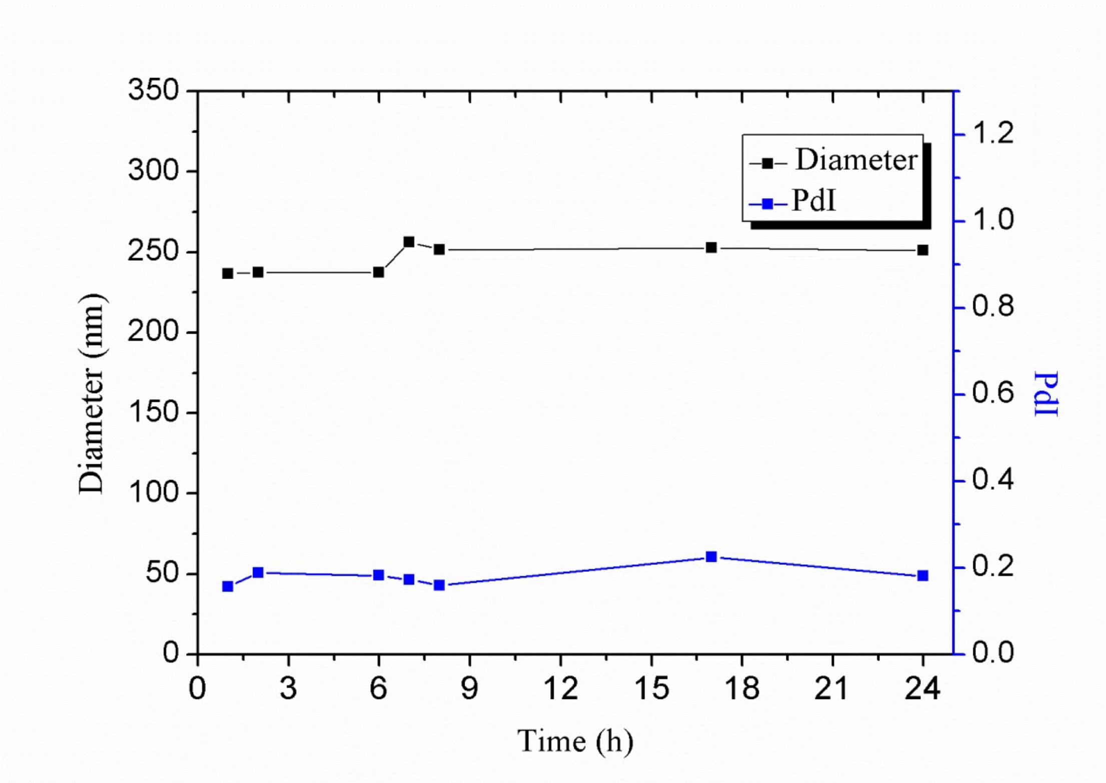

Fig. S2 The changes of the diameter and PdI with different times measured by DLS in PBS with FBS (10 %).

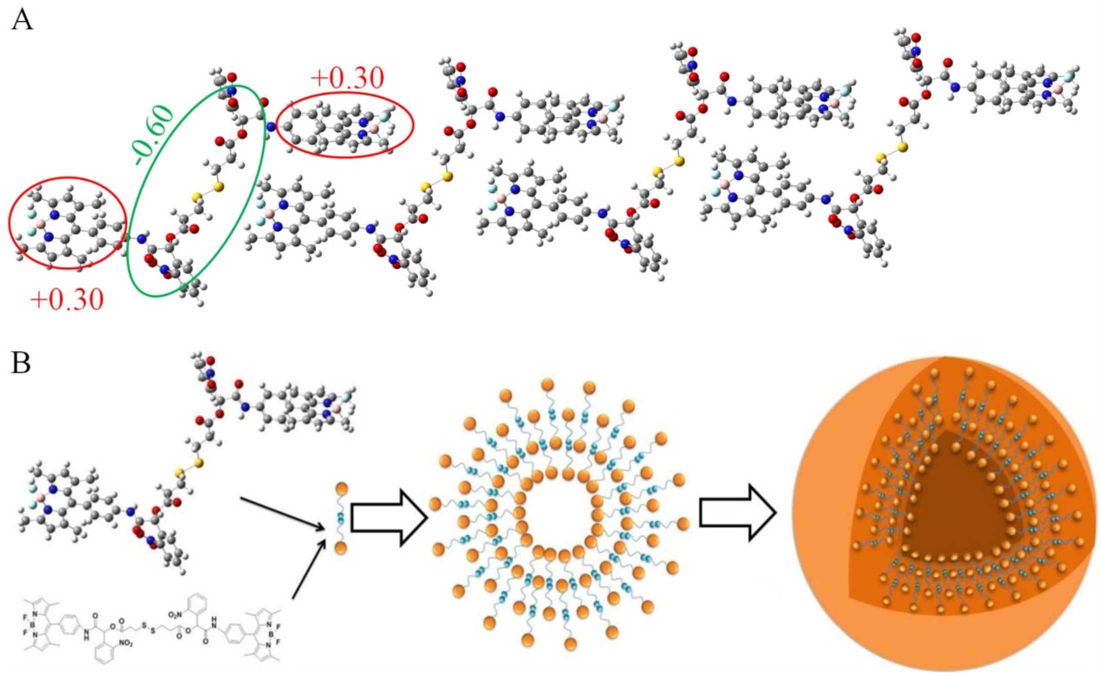

Fig. S3 (A) Ground-state geometrical structure and calculated electron density distribution of SNBDP. (B) Illustration of the formation of supramolecular vesicles.

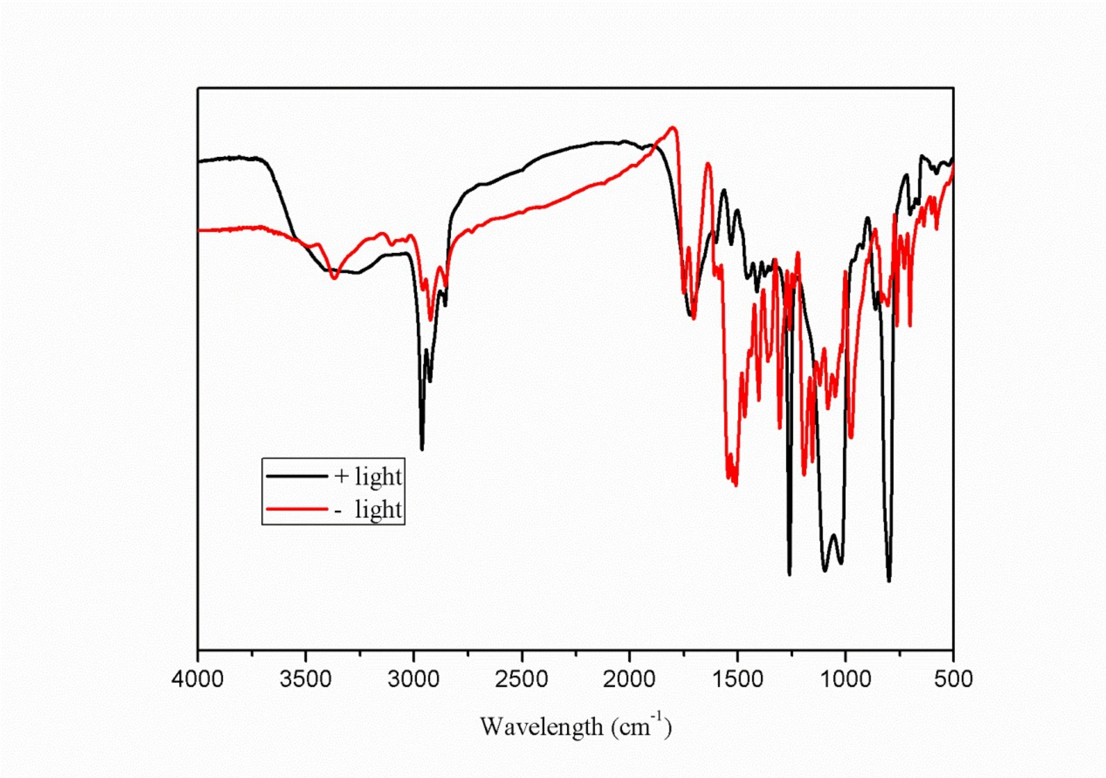

Fig. S4 FT-IR of SNBDP NCs before and after light irradiation.

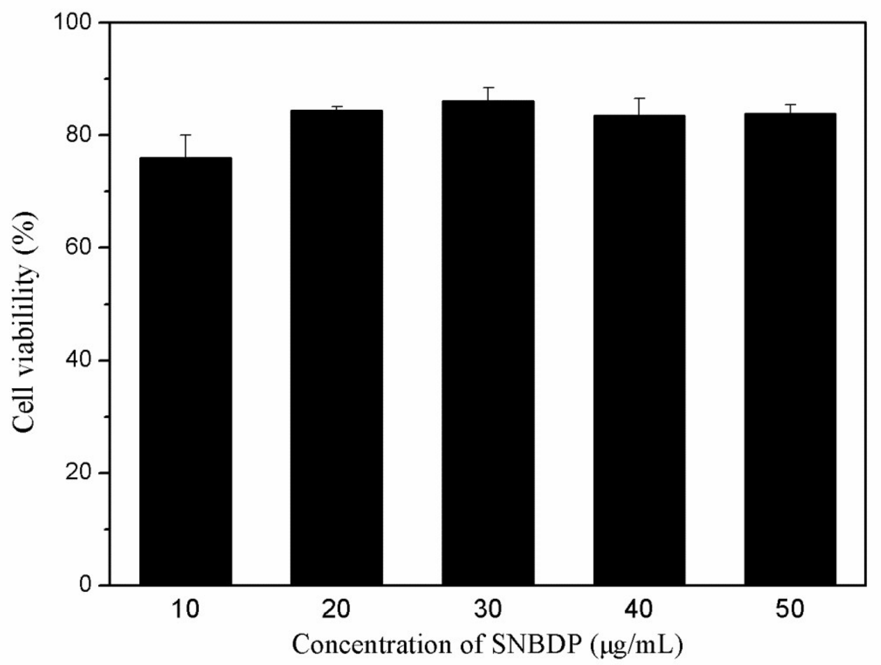

Fig. S5 Cell viability of HeLa cells incubated with SNBDP NCs.

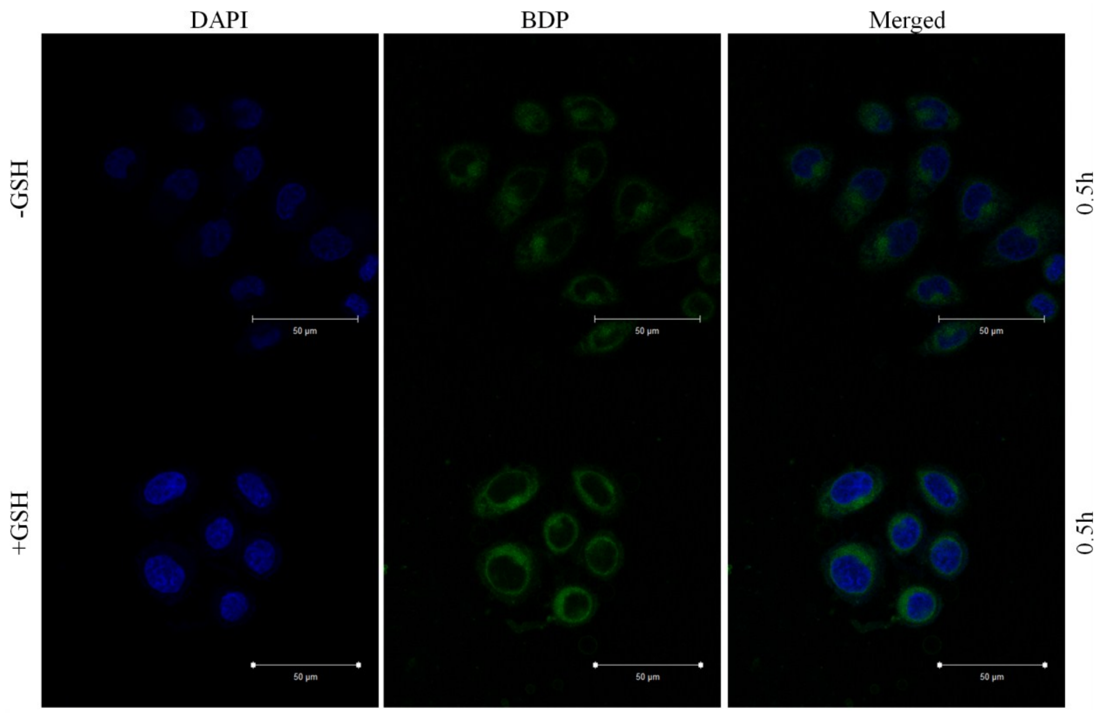

Fig. S6 Representative CLSM images of HeLa cells incubated with SNBDP NCs for 0.5 h without pretreatment and pretreated with 10 mM GSH. For each panel, the

images from left to right show cell nuclei stained by DAPI (blue), BDP fluorescence in cells (green), and overlays of both images. Scale bar, 50 μm.

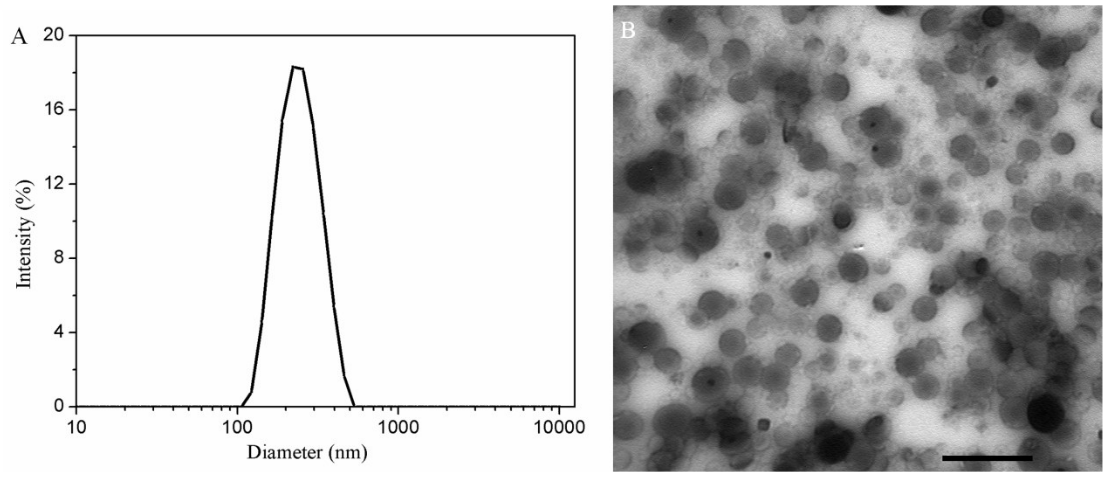

Fig. S7 (A) The size of RhB@SNBDP NCs measured by DLS. (B) TEM images of the RhB@SNBDP NCs. Scale bar, 1 μm.

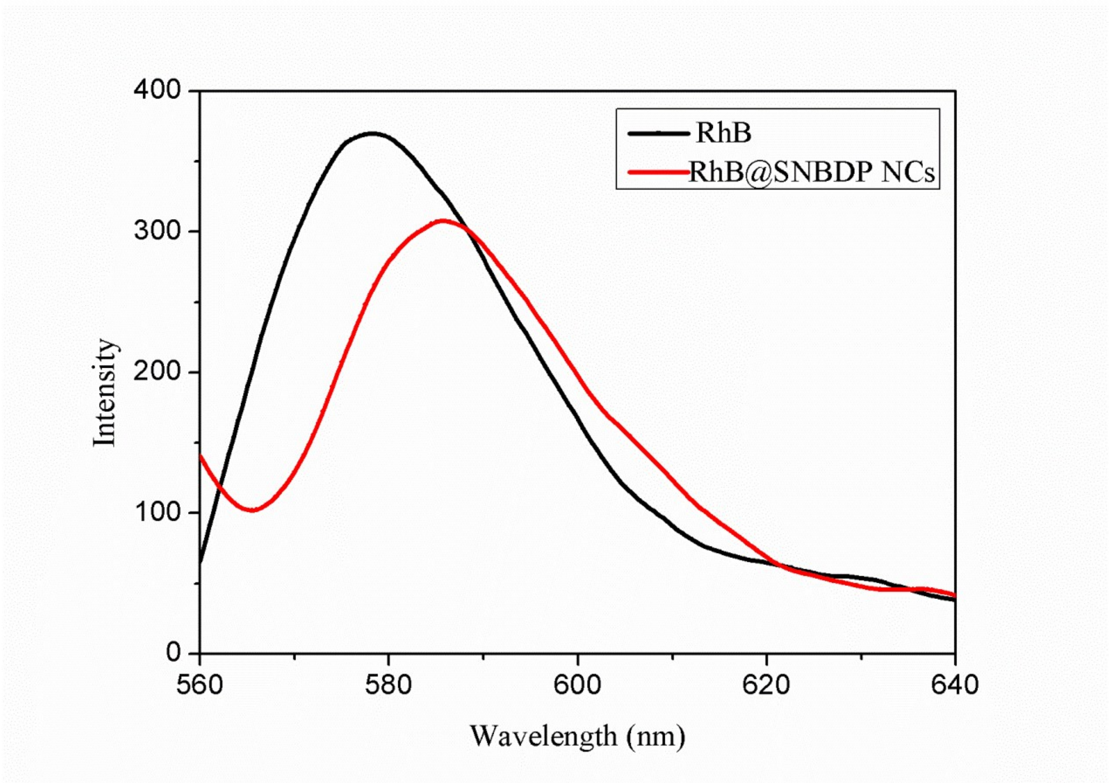

Fig. S8 Fluorescence spectra of RhB@SNBDP NCs and RhB in water.

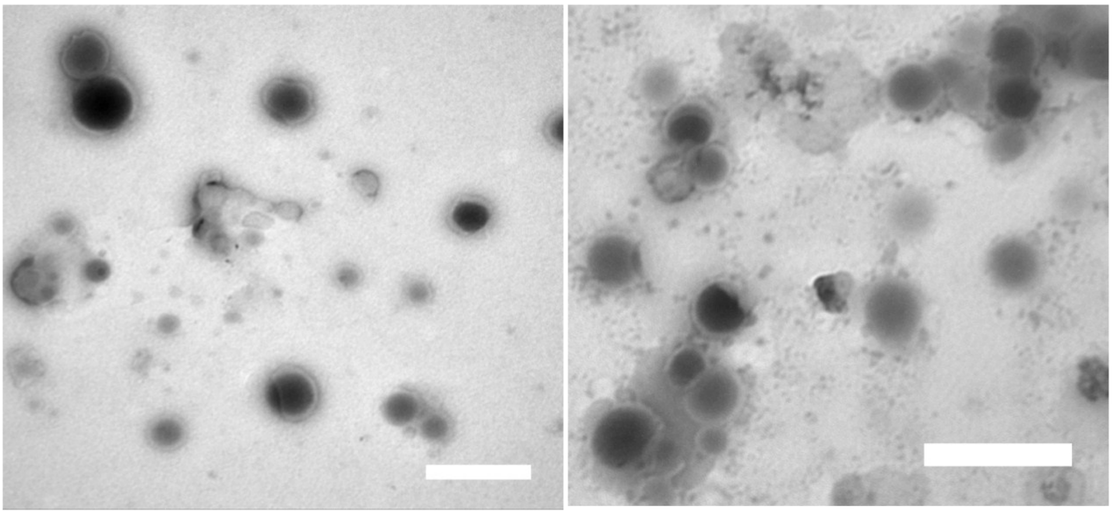

Fig. S9 TEM images of the ICG@SNBDP NCs. Scale bar, 500 nm.

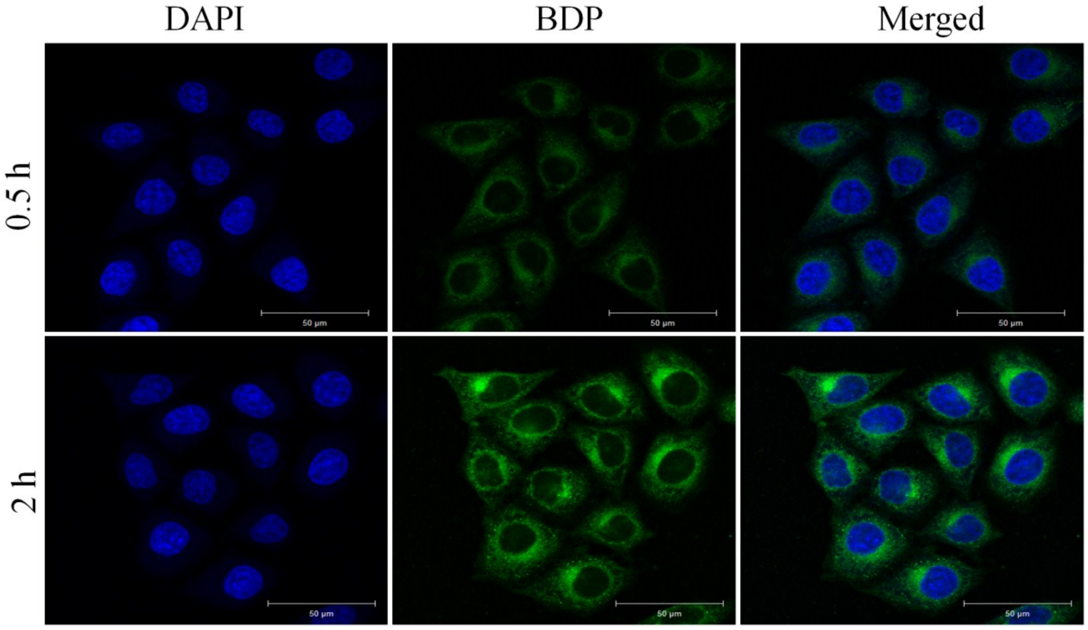

Fig. S10 Representative CLSM images of HeLa cells incubated with ICG@SNBDP NCs for 0.5 h and 2 h. For each panel, the images from left to right show cell nuclei stained by DAPI (blue), BDP fluorescence in cells (green), and overlays of both images. Scale bar, 50 μm.

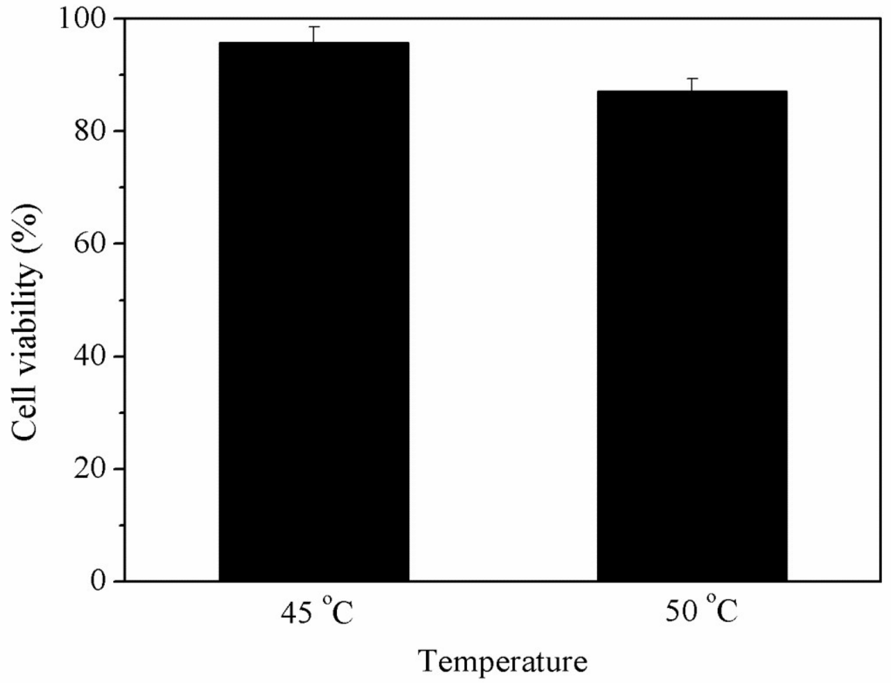

Fig. S11 Relative cell viabilities of HeLa cells after water-bath hyperthermia at 45 ^{o}C and 50 ^{o}C in 5 minutes. All the results were repeated three times and presented as mean ± SD.

Reference:

S1 Frisch, M. J.; Trucks, G. W.; Schlegel, H. B.; Scuseria, G. E.; Robb, M. A.; Cheeseman, J. R.; Scalmani, G.; Barone, V.; Mennucci, B.; Petersson, G. A.; Nakatsuji, H.; Caricato, M.; Li, X.; Hratchian, H. P.; Izmaylov, A. F.; Bloino, J.; Zheng, G.; Sonnenberg, J. L.; Hada, M.; Ehara, M.; Toyota, K.; Fukuda, R.; Hasegawa, J.; Ishida, M.; Nakajima, T.; Honda, Y.; Kitao, O.; Nakai, H.; Vreven, T.; Montgomery Jr., J. A.; Peralta, J. E.; Ogliaro, F.; Bearpark, M.; Heyd, J. J.; Brothers, E.; Kudin, K. N.; Staroverov, V. N.; Keith, T.; Kobayashi, R.; Normand, J.; Raghavachari, K.; Rendell, A.; Burant, J. C.; Iyengar, S. S.; Tomasi, J.; Cossi, M.; Rega, N.; Millam, J. M.; Klene, M. J.; Knox, E.; Cross, J. B.; Bakken, V.; Adamo, C.; Jaramillo, J.; Gomperts, R.; Stratmann, R. E.; Yazyev, O.; Austin, A. J.; Cammi, R.; Pomelli, C.; Ochterski, J. W.; Martin, R. L.; Morokuma, K.; Zakrzewski, V. G.; Voth, G. A.; Salvador, P.; Dannenberg, J. J.; Dapprich, S.; Daniels, A. D.; Farkas, O.; Foresman, J. B.; Ortiz, J. V.; Cioslowski J.; Fox, D. J. GAUSSIAN 09 (Revision B.01), Gaussian, Inc., Wallingford, CT, 2009.
